# Upper Airway Obstruction in a Newborn with Vallecular Cyst

**Published:** 2015-10-01

**Authors:** Birgin Torer, Bilin Cetinkaya, Serkan Yılmaz, Cuneyt Yilmazer, Hande Gulcan

**Affiliations:** 1 Department of Neonatology, Baskent University Faculty of Medicine, Turkey; 2 Department of Otorhinolaryngology, Baskent University Faculty of Medicine, Turkey

**Keywords:** Congenital stridor, Neonate, Premature, Upper airway obstruction, Vallecular cyst

## Abstract

Vallecular cyst is a rare cause of stridor in neonates, which may present as a life threatening airway obstruction. Here, we report a preterm infant with a congenital vallecular cyst who presented with stridor and respiratory distress that developed immediately after birth. She was successfully treated with endoscopic marsupialization.

## CASE REPORT

A female infant, weighing 2020 grams born to a healthy 35-year-old mother at 33 weeks of gestation, was referred because of stridor and respiratory distress developed immediately after birth. On physical examination, she had cyanosis, inspiratory stridor, and tachypnea with suprasternal and subcostal retractions. During intubation, a laryngeal cystic mass at the base of the tongue was noted. Tracheal intubation was done. A flexible fiberoptic nasopharyngolaryngoscopy was performed by the otorhinolaryngologist, which showed a cystic mass arising from laryngeal and lingual mucosa of left epiglottis and obliterating pyriform sinus. Magnetic resonance imaging (MRI) scan revealed a 21x25x18 mm uniloculated cystic mass located behind the tongue; it filled the oropharynx and extended up to the epiglottis (Fig. 1). On day 3, a diagnostic direct laryngoscopy was performed initially, which confirmed a cystic mass in the vallecula; endoscopic marsupialization of the cyst was done (Fig. 2, 3). The neonate required intubation for one-week postoperatively, though the postoperative period was uneventful.

**Figure:**
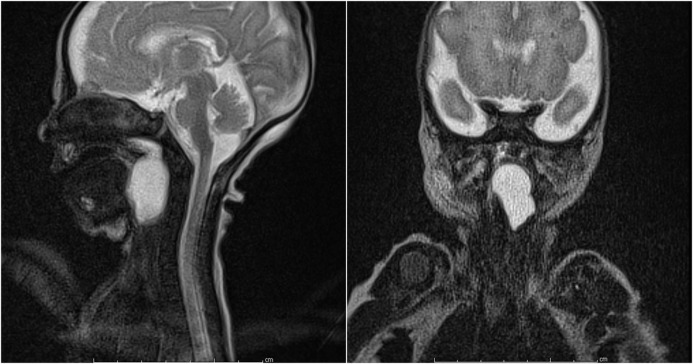
Figure 1: Magnetic resonance images demonstrating the vallecular cyst.

**Figure:**
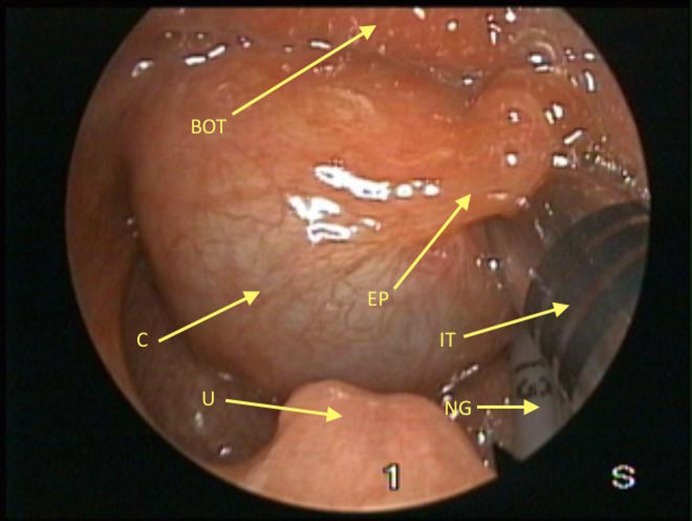
Figure 2: Preoperative endoscopic view of the larynx (BOT; Base of tongue, EP; Epiglottis, C;Cyst, U;Uvula, ET; Endotracheal tube, NG; Nasogastric tube).

**Figure:**
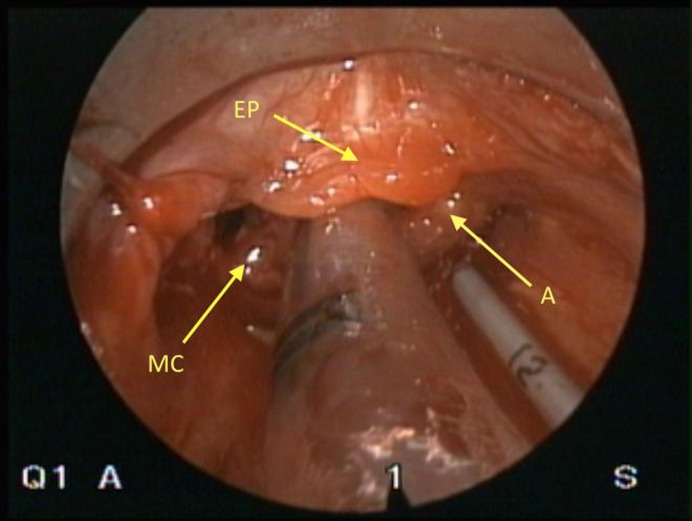
Figure 3: Endoscopic view of the larynx after marsupialization (MC; Marsupialized Cyst, A;Arytenoid cartilage, EP; Epiglottis).

Histopathological analysis revealed a cyst lined by non-keratinizing squamous epithelium with evidence of chronic inflammation in fibrous tissue. There was no evidence of recurrence at follow-up after one year.

## DISCUSSION

Vallecular cyst is thought to occur as a consequence of either ductal obstruction of mucous glands or an embryologic malformation and presents as inspiratory stridor, respiratory distress, apnea, cyanosis and hoarse cry [1-7]. Majority of affected infants develop symptoms within the first week of life [1, 5]. Our patient developed stridor and respiratory distress immediately after birth and upper airway obstruction was detected during tracheal intubation. Feeding difficulties and failure to thrive may be the presenting symptoms in older children [2, 6, 7]. Flexible fiberoptic endoscopy is the most helpful diagnostic tool in the diagnosis of vallecular cysts [1, 3, 6-9]. It allows immediate dynamic assessment of the larynx in a bedside setting. MRI is a valuable imaging method to determine the extent and content of the lesion [5,7,8]. Thyroglossal cyst, thyroid remnant cyst, dermoid cyst, cystic hygroma, hemangioma, lymphangioma, teratoma should be considered in the differential diagnosis of vallecular cyst [1, 5-9]. Direct laryngoscopy under general anesthesia is the major diagnostic tool for confirming the diagnosis of vallecular cyst [4, 7-9]. Prenatal diagnosis of vallecular cyst with ultrasonography and MRI has been reported and earlier diagnosis allows planning perinatal management [10]. Surgical cyst excision is the treatment of choice for vallecular cysts. Aspiration of the cyst is not advocated because of its high recurrence rate but cyst aspiration may offer prompt relief of upper airway obstruction [4, 5]. Endoscopic marsupialization is the recommended surgical approach in infants [2, 5, 7-10]. Extirpation of the cyst with external approach is preferred for treatment of recurrent cysts but it is more invasive than marsupialization and sometimes requires tracheotomy [2, 8, 9]. Hoarseness and dysphagia are complications of external approach. 

Long-term prognosis of vallecular cyst is generally good with minimal recurrence rate [2, 4, 7-9].

## Footnotes

**Source of Support:** Nil

**Conflict of Interest:** Nil
